# Root Canal Operations

**Published:** 1919-06

**Authors:** 


### ROOT CANAL OPERATIONS

Dr. Elmer S. Best recently read a paper before The First District Dental Society, S.N.Y., in which he presented some of the impressions of a visit to the Mayo Foundation. This paper is reported in detail in *The Journal of the Allied Dental Societies*, December, 1918. In these few words he sums up his position with regard to pulpless teeth: "No teeth must be removed unnecessarily and yet no diseased conditions must be left. If a pulpless tooth and its supporting alveolus can not be rid of pathological conditions in any other way, it must be extracted."

Taking up the question of tooth treatment, Dr. Best has much to say that will be of great interest for all of us. He would have us always make an examination of the tooth and surrounding tissues so as to determine very accurately whether or not a root canal operation is indicated. Having decided upon the treatment of the tooth we ought to carefully remove all decay in the tooth previous to placing the rubber dam in position. Then the teeth that are so isolated by the dam are wiped off with iodine and alcohol. If we have decided to remove the vital pulp by means of an injected anesthetic, then the injection is made previous to placing the rubber dam. With the canal freely opened up a small amount of formalin is applied, on a pledget of cotton, to the pulp tissue. This is for the purpose of toughening the tissue and so facilitate its removal. A suitable barbed broach is selected and used to remove the tissue. As each canal is cleaned of its pulp it is sealed up with oxychloride of zinc and attention directed to the other canals. In this way the possibility oı contaminating the cleared canal is reduced to a minimum. In constricted canals where the finest barbed broach will not go, then try an apex broach, and if necessary use a smooth broach with sodium potassium or sulphuric acid thirty per cent. These solvents will soften the dentinal wall, and the canal may be enlarged by means of broaches. A sterile paper point is then inserted and sealed in until all the canals are made ready for the filling. If the X-ray picture shows this part of the work to have been done satisfactorily, then the permanent filling may be gone on with at once. If, however, the operation can not be completed at this sitting, seal in the canals a very mild dressing of oil of cloves, covering it securely with oxychloride of zinc cement, and moisten the cavity with xylol and fill with temporary stopping. (Xylol may be used very satisfactorily to remove gutta percha from incompletely filled root canals.)

Greater care is required in preparing teeth for filling in which there are dead, gangrenous pulps. It is best to culture the canals and wash cautiously with one per cent. chlorosene, using a pulp canal syringe. Formo-cresol is then sealed in. At the next sitting the dressing is removed and sodium potassium used in the canal, taking care not to enter the apical third. This solution is then neutralized with bichloride of mercury and the entire canal dried carefully. Formo-cresol dressings are renewed from time to time until the canals are free from bacteria.

For sealing up the foramina, a piece of gutta percha cone is condensed into the apex by moistening with rosin and chloroform. Just at this time we are free to remove all infected dentine from the canals and also effect their enlargement. This is best accomplished with heavy barbed broaches and Kerr files. Chlora percha is now used to complete the canal filling and oxychloride of zinc cement to seal over the orifice.

Recently Dr. Howe advocated the use of silver nitrate in root canal work, and its use has presented some difficulties to at least a few operators. For the benefit of these men Walter F. Provan, D.M.D., of Boston, offers, a few suggestions through the *Dental Cosmos.* He finds that satisfactory results in the use of this solution are obtained most easily if the canals are thoroughly cleaned out before attempting to make the application. Again, it is very necessary to get the solution (solution No. 1) to the apex. Capillary attraction will not take it there if the root canal is dry, because air will be caught in the upper end of the canal. Difficulty is experienced in attempting to dislodge this air with a smooth broach, and there is the added danger of lacerating the tissue beyond the end of the root. To avoid this dip a cotton point (Johnson and Johnson) in solution No. 1 and insert it in the canal, forcing the point well up to the apex. Owing to the softness of the point the tissues can not easily be harmed. It is possible at the same time to moisten the walls of the canal with the solution. Next, a drop of the solution is introduced into the pulp chamber with a pair of cotton pliers. With a smooth broach work the solution to the apex of the canal. Allow it to remain for three or four minutes, and then follow with a drop of No. 2 solution. Again wait three or four minutes. The canal is now dried out slightly with a cotton point and again a drop of solution No. 1 is inserted. This is followed by a drop of eugenol, the mixture being worked into the canal by means of a smooth broach. After a few minutes all excess moisture is dried out with cotton points and some chloro percha introduced until the root canal is full. A packing of gutta percha points completes the filling. Cement is poured over the canal opening and all is then ready for the tooth filling.

The advantage claimed for eugenol by Dr. Provan is that it prevents soreness following the use of the silver nitrate solution in the canals. In a few cases he is prepared to admit soreness may occur, and explains it as follows: "The precipitation which follows the addition of solution No. 2 and eugenol to solution No. 1 seals the apical foramina if they are small, and if they are large the gutta percha filling does it. This precipitation effectually sterilizes the root canal, and with the gutta percha filling cuts off any subsequent re-infection from this source; but there is an apical infection existing which has not yet been dealt with, and nature has to clear up this infected region. The vent for gas or pus has been sealed, and unless these can be absorbed as fast as they are formed, soreness is sure to develop. If this kind of trouble arises and pus forms, do not ream out the root canal. A lancet thrust will give relief, and the case will clear up of itself.—*Oral Health Compendium.*

